# Assessment of spatial and temporal variations in trace element concentrations using honeybees (*Apis mellifera*) as bioindicators

**DOI:** 10.7717/peerj.5197

**Published:** 2018-07-16

**Authors:** Nenad M. Zaric, Isidora Deljanin, Konstantin Ilijević, Ljubiša Stanisavljević, Mirjana Ristić, Ivan Gržetić

**Affiliations:** 1 Innovation Center of the Faculty of Technology and Metallurgy, Belgrade, Serbia; 2 Faculty of Chemistry, University of Belgrade, Belgrade, Serbia; 3 Faculty of Biology, University of Belgrade, Belgrade, Serbia; 4 Faculty of Technology and Metallurgy, University of Belgrade, Belgrade, Serbia

**Keywords:** Biomonitoring, Honeybees, Trace metals, Pollution, Monitoring, Bioindicator

## Abstract

With the increase in anthropogenic activities metal pollution is also increased and needs to be closely monitored. In this study honeybees were used as bioindicators to monitor metal pollution. Metal pollution in honeybees represents pollution present in air, water and soil. Concentrations of As, Cs, Hg, Mo, Sb, Se, U and V were measured. The aim of this study was to assess spatial and temporal variations of metal concentrations in honeybees. Samples of honeybees were taken at five different regions in Serbia (Belgrade - BG, Pančevo - PA, Pavliš - PV, Mesić - MS, and Kostolac - TPP) during 2014. Spatial variations were observed for Sb, which had higher concentrations in BG compared to all other regions, and for U, with higher concentrations in the TPP region. High concentrations of Sb in BG were attributed to intense traffic, while higher U concentrations in the TPP region are due to the vicinity of coal fired power plants. In order to assess temporal variations at two locations (PA and PV) samples were taken during July and September of 2014 and June, July, August and September of 2015. During 2014 observing months of sampling higher concentrations in July were detected for Sb and U in BG, which is attributed to lifecycle of plants and honeybees. During the same year higher concentrations in September were observed for As, Sb in PA and Hg in PV. This is due to high precipitation during the peak of bee activity in spring/summer of 2014. No differences between months of sampling were detected during 2015. Between 2014 and 2015 statistically significant differences were observed for Hg, Mo and V; all elements had higher concentrations in 2014. This is in accordance with the trend of reduction of metal concentrations in the bodies of honeybees throughout the years in this region.

## Introduction

Metal pollution can be of natural and anthropogenic origin. Due to industrialization in the last few decades human activities have become a primary source of metal pollution in the environment (Johnson, 2015). Metals in soil can have natural origin, namely from the parent rock, but anthropogenic activities can be the primary source. Anthropogenic sources of metals can be traffic, industry, energy production (thermal power plants fired by coal), intense agriculture, etc. This pollution can be spread throughout the environment, including air, water and soil, and thus have an impact on living organism (Fargašová, 2001; Diels, Van der Lelie & Bastiaens, 2002; Shanker et al., 2005).

Due to its mostly negative impact on living organisms, metal pollution needs to be constantly monitored. Most of the classical monitoring methods require expensive equipment or cover just a small area of interest. Consequently, different plant species have been proposed as bioindicators of metal pollution (Akguc et al., 2010; Serbula et al., 2013; Deljanin et al., 2016). Considering that plants are stationary organisms, they cover only a small area in close proximity of the plant. Therefore different animal species have been recently considered as bioindicators (Loflen, 2013; García-Hernández et al., 2015; Jovičić et al., 2016; Flache et al., 2017).

Honeybees cover large areas during their foraging activities. Each forager bee completes 12-15 foraging trips per day, flying up to 10 km away from the colony and covering an area of approximately 7 km^2^ (Perugini et al., 2011; Hladun et al., 2013; Johnson, 2015; Van der Steen et al., 2016). During these foraging activities, honeybees can accumulate metals from the environment (Johnson, 2015). Honeybees accumulate metals by flying through air and collecting suspended particulate matter (PM) on their hairy bodies. They also ingest metals through water they drink or by using water to cool down the hive. By visiting flowers they can collect metals from pollen and nectar transported into plant from soil. Airborne PM can also be deposited on flowers, as well as soil dust from topsoil erosion (Hladun et al., 2013). Honeybees in contact with flowers collect these particles on their hairy bodies (Negri et al., 2015). Therefore, metal pollution in honeybees represents pollution present in air, water and soil (Sadeghi et al., 2012; Hladun, Parker & Trumble, 2015; Zarić et al., 2017). Honeybees (*Apis mellifera*) have already been used as bioindicators in the past few decades (Bromenshenk et al., 1985; Leita et al., 1996; Porrini et al., 2002; Gutiérrez et al., 2015; Zarić et al., 2016; Giglio et al., 2017). They have been used to detect metal pollution in different environments including urban and rural regions (Porrini et al., 2002; Perugini et al., 2011); industrial areas (Van Der Steen, De Kraker & Grotenhuis, 2012); and protected areas (Ruschioni et al., 2013). Through the study of soil, air, plants and honeybees from the same region it was concluded that bees can be used to assess anthropogenic influence in the environment (Zhelyazkova, 2012; Zhou et al., 2018).

The aim of this study was to use honeybees as bioindicators to monitor heavy metal pollution in Serbia. Concentrations of As, Cs, Hg, Mo, Sb, Se, U and V were measured in bodies of forager bees at five locations with different anthropogenic activities. At two locations sampling was performed for two years 2014 and 2015 in the months of June, July, August and September to test annual and seasonal variations. This is the first time that honeybees have been used to determine concentration of Cs and U.

## Materials and Methods

### Sampling regions

There were five different sampling regions. Each region had at least one apiary from which the samples were taken. At each apiary at least two colonies were sampled.

#### Belgrade (BG)

**Figure 1:**
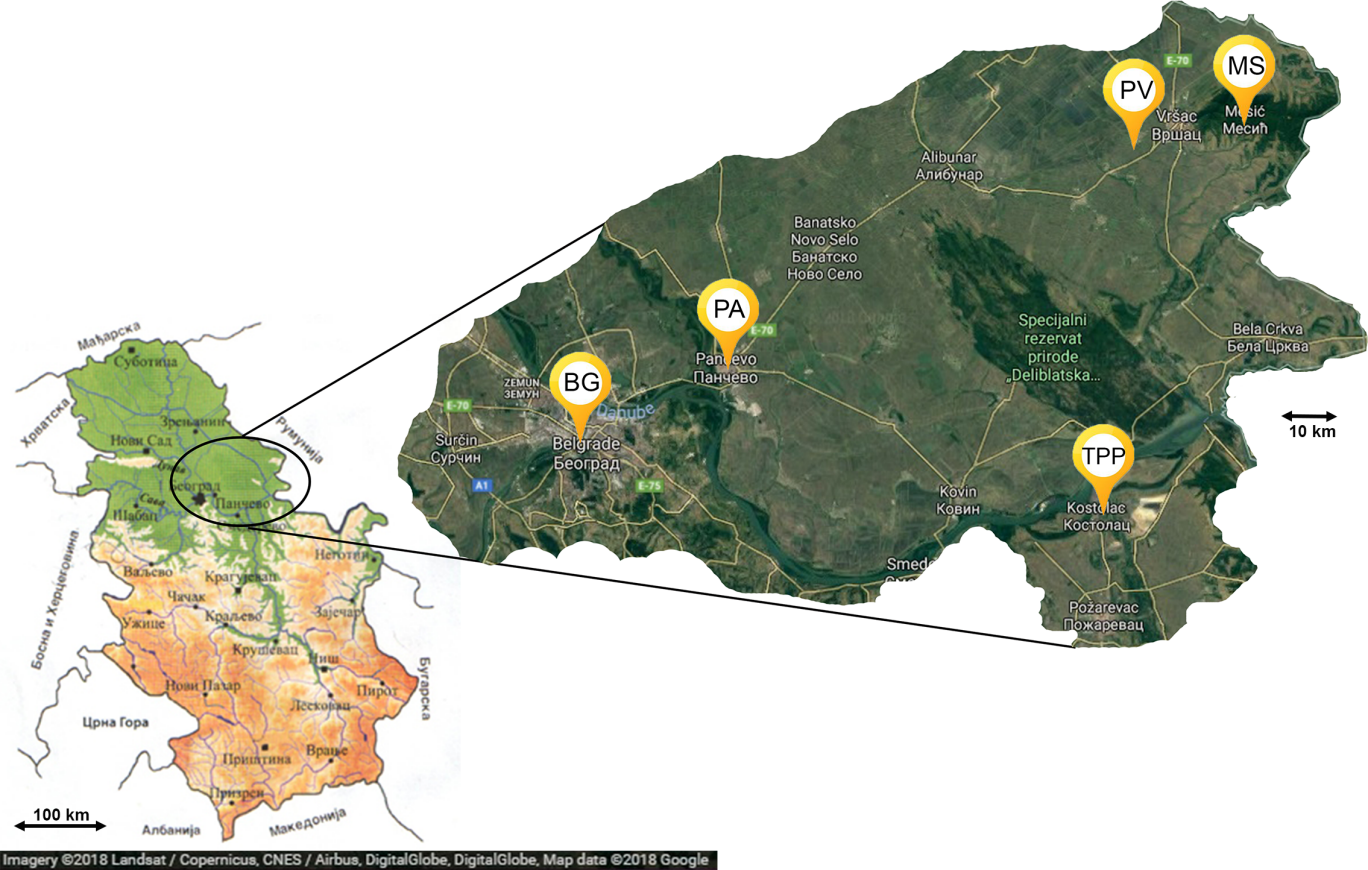
Map of sampling locations (Map data: Google, DigitalGlobe). BG, Belgrade; PA, Pančevo; PV, Pavliš; MS, Mesić; TPP, Kostolac

Belgrade is the capital of Serbia. There were two apiaries in the city of Belgrade, both in the urban center (Fig. 1). One was located at the Faculty of Veterinary Medicine—University of Belgrade, while the other was at the Faculty of Agriculture—University of Belgrade.

#### Pančevo (PA)

The city of Pančevo is the center of the South Banat district, Vojvodina province, Serbia. It is well known as an industrial city in which most of countries’ petrochemical industry is located. Samples were taken from two apiaries, one in the eastern part of the city and one in the western. The apiaries were located so that the bees flying form them would be influenced by two major pollution sources in the city, fertilizer production plant and oil refinery.

#### Pavliš (PV)

The village of Pavliš is suburban area, located at the outskirts of the second largest city in South Banat district, Vršac. There is no heavy industry in the vicinity of the village. The main industry present is pharmaceutical company located in Vršac, close to the village. One apiary was sampled.

#### Mesić (MS)

Mesić is a small village bordering a protected area. The village is in a rural area, surrounded by agricultural area from three sides with the protected area to the north. There was one apiary from where samples were taken.

#### Kostolac (TPP)

There were three apiaries located in the municipality of Kostolac. This region is characterized by presence of two thermal power plants (TPP), Kostolac A and B. One apiary was in the town of Kostolac in the vicinity of TPP Kostolac A. Second apiary was in the village Drmno, located near TPP Kostolac B. This village is surrounded by the open pit coal mine “Drmno”. Third apiary was in between the two TPPs in the village of Stari Kostolac, close to the ash disposal site.

### Sampling

During 2014 samples from Mesić were taken in July from two colonies in one apiary. In Belgrade samples were taken from two colonies at each of the two apiaries in July, while in September samples were taken from one colony at each apiary. In TPP region samples were taken from one colony at apiaries located in Kostolac and Drmno, and from two colonies at apiary in Stari Kostolac in July and from one colony at each of the three apiaries in September. In Pančevo both in July and September samples were taken from two colonies at both apiaries. In Pavliš in July and September samples were taken from two colonies at one apiary. Samples were not taken from location MS in September (Table 1).

**Table 1:** Sampling locations with year/month of sampling.

Sampling location	Year/month	
	2014	2015
BG	July	–
	September	–
TPP	July	–
	September	–
MS	July	–
PA	–	June
	July	July
	September	September
PV	–	June
	July	July
	–	August
	September	September

For the 2015 sampling campaign two locations were chosen: Pančevo and Pavliš. These locations were chosen to represent two locations with different human activities having different environmental impacts. Pančevo represented an industrial city with petrochemical industry and oil refinery, and Pavliš represented a suburban area not influenced by industrial or traffic pollution. In Pančevo there were two apiaries and honeybees were sampled from one colony at each apiary in Jun, July and September. In Pavliš there was one apiary, where samples were taken from two colonies in June, July, August and September (Table 1).

The samples were taken on the same day from each apiary. Forager bees were collected directly from the outer forage frame, containing bees but no brood (Van Der Steen, De Kraker & Grotenhuis, 2012). It is documented that adult forager honeybees, specialized in gathering of nectar and pollen, are located at the outer frame of the hive (Bilalov et al., 2015). About 100 of worker bees, weighing aproximatelly10g, were taken and transferred directly into sterile plastic containers. The bees were killed in the laboratory by freezing at −21 ± 3 °C. Considering that there are approximately 20 to 80 thousand worker bees in each colony, our experiment did not influence the survival of the bee colony.

### Sample preparation and analysis

Samples of honeybees were measured and dried in the oven at 60 °C until constant mass (cca. 96h). Aliquot of each dried sample around 0.5000 g was measured and digested according to the US EPA SW-846 Method 3052, in closed Teflon vessels, under high pressure, with 7 ml of concentrated HNO_3_ (p.a.) and 2 ml of concentrated H_2_O_2_ (p.a.). Digestion was performed in a closed microwave digestion system (ETHOS 1, Advanced Microwave Digestion System, Milestone, Italy) by gradually heating the samples up to 200 °C (15 min), followed by another 15 min at the same temperature. Each sample was cooled, transferred to a 25-mL volumetric flask and diluted to 25 mL with deionized water.

The concentrations of As, Cs, Hg, Mo, Sb, Se, U and V were determined by the inductively coupled plasma mass spectrometry (ICP-MS) using Agilent 7500ce instrument equipped with Octopole Reaction System (ORS) in FullQuant mode. The measurements for every sample were done in three replicates and the average values were calculated for every sample. Calibration of ICP-MS was performed using Multielement Standard Solution IV (Fluka) with six standard solutions. Standard solutions and blanks were prepared in 2% HNO_3_. Tuning solution containing 1 µg l^−1^ Li, Mg, Co, Y, Ce and Tl (Agilent, Santa Clara, CA, USA) was used for the instrument optimization. Accuracy was in the range 94–108% of the certified reference material SLRS-6 (river water certified reference material for trace metals and other constituents, National Research Council of Canada).

LOD were 0.015 mg kg^−1^, 0.002 mg kg^−1^, 0.01 mg kg^−1^, 0.07 mg kg^−1^, 0.01 mg kg^−1^, 0.083 mg kg^−1^, 0.001 mg kg^−1^, and 0.001 mg kg^−1^ for As, Cs, Hg, Mo, Sb, Se, U and V respectively.

### Data analysis

Average values and standard deviations were calculated for every region. Normality of the data was checked using Kolmogorov–Smirnov test. For some elements, the data was not normally distributed. Such data was log-transformed prior to further statistical analysis. Statistically significant differences between locations, year or month of sampling were determined using one-way ANOVA, followed by Tukey’s post hoc test (differences were considered significant if *p* ≤ 0.05). For statistical analysis, in samples where the elements concentrations were below LOD the used values were one half of LOD.

## Results

Average metal concentrations ±standard deviations for 2014 are given in Table 2. Most of the analyzed elements were above the limit of detection (LOD) during 2014, except Sb at location PV during July (Table S1). In 2015, Hg was below the LOD in most of the analyzed samples. Elements that also had concentrations below the limit of detection were Sb, Se and U (Table 3).

**Table 2:** Mean metal concentrations (mg kg^−1^) and standard deviations in bees taken from five locations during 2014 (*N* = 27).

		Element						
Location	As	Cs	Hg	Mo	Sb	Se	U	V
BG	0.28 ± 0.16	0.0267 ± 0.0054	0.070 ± 0.032	0.85 ± 0.28	0.052 ± 0.019	0.23 ± 0.10	0.0055 ± 0.0033	0.232 ± 0.063
PA	0.206 ± 0.089	0.031 ± 0.012	0.118 ± 0.074	0.57 ± 0.20	0.025 ± 0.010	0.246 ± 0.082	0.0027 ± 0.0014	0.254 ± 0.072
PV	0.19 ± 0.13	0.0133 ± 0.0028	0.115 ± 0.080	0.83 ± 0.34	0.016 ± 0.010	0.206 ± 0.097	0.0030 ± 0.0011	0.193 ± 0.061
MS	0.195 ± 0.036	0.026 ± 0.012	0.026 ± 0.036	0.304 ± 0.024	0.0219 ± 0.0024	0.093 ± 0.021	0.00521 ± 0.00015	0.1155 ± 0.0058
TPP	0.40 ± 0.24	0.053 ± 0.038	0.045 ± 0.040	0.90 ± 0.99	0.0206 ± 0.0036	0.39 ± 0.12	0.0151 ± 0.0063	0.54 ± 0.32

**Table 3:** Mean metal concentration (mg kg^−1^) and standard deviations in bees from PA and PV taken in June, July, August and September 2015 (*N* = 14).

		Element							
Location	Month	As	Cs	Hg	Mo	Sb	Se	U	V
PA	Jun	0.026 ± 0.021	0.020 ± 0.012	<LOD	0.233 ± 0.011	0.0136 ± 0.0053	<LOD	0.001135 ± 0.000042	0.053 ± 0.028
	July	0.17 ± 0.13	0.64 ± 0.80	0.012 ± /	0.53 ± 0.40	<LOD	0.1243 ± 0.0015	0.0072 ± 0.0093	0.045 ± 0.023
	August	–	–	–	–	–	–	–	–
	September	0.045 ± 0.016	0.031 ± 0.015	<LOD	0.214 ± 0.019	<LOD	<LOD	<LOD	0.03045 ± 0.00036
PV	Jun	0.25 ± 0.10	0.024 ± 0.011	0.0119 ± /	0.53 ± 0.15	0.47 ± 0.64	0.38 ± 0.12	0.00301 ± 0.00099	0.194 ± 0.037
	July	0.241 ± 0.013	0.028 ± 0.014	–	0.364 ± 0.043	0.0182 ± 0.0067	0.314 ± 0.012	0.0103 ± 0.0059	0.23 ± 0.21
	August	0.113 ± 0.019	0.0279 ± 0.0036	–	0.29 ± 0.13	0.0205 ± 0.0038	0.236 ± 0.031	0.00200 ± 0.00067	0.092 ± 0.075
	September	0.082 ± 0.012	0.114 ± 0.091	<LOD	0.44 ± 0.17	<LOD	0.258 ± 0.019	0.0051 ± 0.0034	0.100 ± 0.025

Most abundant element in bodies of honeybees at all locations was Mo, while U had the lowest concentrations. The rest of the elements were ranked in the descending order: in BG As>Se>V>Hg>Sb>Cs; PA Se>V>As>Cs>Hg>Sb; PV Se>As>V>Sb>Hg>Cs; As>V>Se>Hg>Cs>Sb; and TPP region V>As>Se>Cs>Hg>Sb.

During 2014 regardless of the sampling month, ANOVA showed statistically significant differences between at least two locations for Sb, Se, U and V. Namely, statistically higher concentrations in BG compared to other locations was observed for Sb (*F* = 8.56, *p* = 0.003). TPP region had statistically higher concentrations of U compared to other locations (*F* = 12.26, *p* = 0.0001), and higher concentrations of Se (*F* = 5.15, *p* = 0.0056) and V (*F* = 4.01, *p* = 0.0183) compared to MS.

Statistically significant differences between PA and PV locations were observed for Se (*F* = 40.01, *p* = 0.0001) and V (*F* = 6.12, *p* = 0.0293), with higher concentrations in PV samples.

During 2014 samples were taken in July and September at five locations. In BG statistically significant differences were observed for Sb (*F* = 9.38, *p* = 0.0375) and U (*F* = 13.84, *p* = 0.0205), with higher concentrations in July. Higher concentrations in September compared to July were observed in PA for As (*F* = 11.73, *p* = 0.141) and Sb (*F* = 6.04, *p* = 0.0493), and in PV for Hg (*F* = 33.78, *p* = 0.0283). Looking at differences between month of July and September 2014 for all locations together no significant differences were observed. Samples from MS were taken only in July. There were no statistically significant differences between the sampling months in TPP region. As for 2015, ANOVA showed that there were no statistically significant differences in monthly concentrations of the analyzed elements.

For locations PA and PV, statistically significant differences in metal concentrations, between two years of sampling, 2014 and 2015, were observed for Hg (*F* = 26.64, *p* = 0.0001), Mo (*F* = 9.97, *p* = 0.0043) and V (*F* = 13.63, *p* = 0.0011). All these elements had higher concentrations during 2014 compared to 2015 (Table 4).

**Table 4:** Mean metal concentrations (mg kg^−1^) and standard deviations in bees collected at PA and PV locations in 2014 and 2015 (*N* = 26).

		Element							
Location	Year	As	Cs	Hg	Mo	Sb	Se	U	V
PA	2014	0.206 ± 0.089	0.031 ± 0.012	0.118 ± 0.074	0.57 ± 0.20	0.025 ± 0.010	0.246 ± 0.082	0.0027 ± 0.0014	0.254 ± 0.072
	2015	0.079 ± 0.089	0.23 ± 0.48	0.0057 ± 0.0031	0.32 ± 0.24	0.0104 ± 0.0036	0.09 ± 0.030	0.0035 ± 0.0058	0.043 ± 0.019
PV	2014	0.19 ± 0.13	0.0133 ± 0.0028	0.115 ± 0.080	0.83 ± 0.34	0.016 ± 0.010	0.206 ± 0.097	0.0030 ± 0.0011	0.193 ± 0.061
	2015	0.172 ± 0.089	0.048 ± 0.053	0.0102 ± 0.0024	0.41 ± 0.14	0.13 ± 0.32	0.296 ± 0.076	0.0051 ± 0.0043	0.15 ± 0.11

## Discussion

From the obtained results it can be observed that at all locations the most abundant element in honeybees was Mo, while U had the lowest concentrations. Other elements had different rank orders depending on location. This is most likely due to different anthropogenic activities.

### Spatial variations

Higher Sb concentrations in urban regions has been reported in earlier studies (Manta et al., 2002; Yongming et al., 2006). Concentrations of Sb in urban environments has been linked to intense traffic, specifically to the use of brakes (Thorpe & Harrison, 2008). Antimony was detected in brake dust, since manufacturers use Sb_2_S_2_ instead of asbestos in brake linings (Jang & Kim, 2000; Von Uexküll et al., 2005).

Considering that the results showed higher concentrations of U, Se and V in the area impacted by thermal power plants Kostolac A and Kostolac B, it can be assumed that the elements originate from coal combustion occurring in these power plants. It was already pointed out that these power plants are sources of high concentrations of Al, Cr and Fe found in the region (Zarić et al., 2016; Zarić et al., 2018). Earlier studies showed that U comes from the combustion of coal in thermal power plants (Sengupta & Agrahari, 2017).The same was observed for coal fired power plants in Serbia (Životić et al., 2008; Ćujić et al., 2015). The study by Životić et al. (2008) also stated that non-volatile elements like U are mostly retained in solid combustion waste. The TPP region also contains an ash disposal site, which implicates that higher concentrations of U detected in honeybees from this region are the result of coal combustion in thermal power plants Kostolac A and Kostolac B. Since the concentrations of Se and V in TPP region are higher only in comparison to MS it can be suggested that these elements do not originate only from coal combustion.

### Temporal variations

In BG higher concentrations of Sb and U in July compared to September of 2014 is most likely due to ecology of the honeybees and plant lifecycle. In July more plants bloom and honeybees more frequently fly out of the hive to collect pollen and nectar (Zarić et al., 2016). With the reduction of amount of pollen and nectar in the autumn (September) brood rearing is decreased, and with it the proportion of older bees in the colony. This leads to larger amount of younger bees in the sample that have been less exposed to environmental pollution (Kauffeld, 1980).

Higher concentrations in September compared to July in 2014 in PA and PV for As, Sb, and Hg, respectively, can be explained by extreme weather conditions during the months of intense bee activity. During 2014 PA and PV had extreme rain and biggest floods in the last 100 years (Tošić, Unkašević & Putniković, 2017; Zarić et al., 2017). Earlier studies concluded that wet weather has an impact on the reduction of metal concentrations in honeybees (Lambert et al., 2012; Satta et al., 2012).

Although for most of the samples taken in 2014 there were no statistically significant differences between the months of the sampling it can be observed that the concentrations are higher in July, which was explained by the ecology of honeybees and life cycle of plants (Table S1).

To better study temporal variation in metal concentrations, two locations, PA and PV, were selected out of the five previously sampled. At these locations samples were taken in June, July, August and September 2015. ANOVA statistical analysis was performed on these samples. Each location was tested separately, as well as with the other location to check whether there were significant differences between different months of sampling. The results showed no statistically significant differences whatsoever. Standard deviations are high due to the nature of honeybees (Formicki et al., 2013; Zarić et al., 2016). This could be the reason behind the lack of statistically significant differences, although differences in concentrations between months can be observed in Table 3.

Also, metal concentrations in 2014 and 2015 were compared to check if there are differences between the years of sampling. Statistically significant differences were observed; Hg, Mo and V have higher concentration in 2014. It should be mentioned that Hg was below the LOD for all of the samples in 2015, except for location PV in June, so this affected the findings. In an earlier study it was concluded that metal concentration in honeybees from Serbia are in decline throughout the years (Zarić et al., 2017). The present study confirms previous conclusions.

### Comparison with literature data

For the analyzed elements available literature data together with data from this study is presented in Table 5. For Cs and U there is no literature data available. To the best of our knowledge this is the first time that concentrations of Cs and U in bodies of honeybees are reported.

**Table 5:** Range and average concentrations (mg kg^−1^) of metals in bodies of adult honeybees reported in this study and in the literature.

Element	Current study (range and average concentrations)	Earlier studies (range and average concentrations)	
As	<0.015–0.74 (0.22)	0.027–0.05 (–)	Giglio et al. (2017)
		0.017–0.068 (0.04)	Sadeghi et al. (2012)
		0.67–0.83 (0.71)	Van Der Steen, De Kraker & Grotenhuis (2012)
Cs	0.0099–1.21 (0.065)	–	–
Hg	<0.01–0.25 (0.07)	ND	Giglio et al. (2017)
Mo	0.20–3.14 (0.61)	0.35–5.28 (0.75)	Van der Steen et al. (2016)
		0.36–1.16 (0.62)	Van Der Steen, De Kraker & Grotenhuis (2012)
Sb	<0.005–0.93 (0.045)	0.13–3.22 (0.31)	Van der Steen et al. (2016)
		0.09–0.19 (0.11)	Van Der Steen, De Kraker & Grotenhuis (2012)
Se	<0.083–0.58 (0.24)	0.00–0.76 (0.12)	Dżugan et al. (2018)
		0.77–4.37 (2.10)	Van der Steen et al. (2016)
		1.15–1.53 (1.28)	Van Der Steen, De Kraker & Grotenhuis (2012)
		1.84–5.98 (3.09)	Roman (2005)
U	<0.001–0.024 (0.006)	–	–
V	0.02–1.0 (0.24)	0.06–0.13 (–)	Giglio et al. (2017)
		0.01–0.32 (0.04)	Van der Steen et al. (2016)
		0.006–0.31 (0.2)	Van Der Steen, De Kraker & Grotenhuis (2012)

Range and average concentrations of most analyzed trace elements are in the ranges reported in the literature. Only Sb average concentrations in this study are considerably lower than those reported in two studies by Van der Steen et al. (2012) and Van der Steen et al. (2016), although they have the same range.

### Factors influencing metal accumulation by honeybees

There are a number of factors that could influence the level of metal accumulation by honeybees. The most important factor is certainly the levels of metals present in the environment surrounding the honeybee colony. But regardless of levels of metals there factors including health of the colony and individual bees and their age that can also influence metal accumulation.

Healthy colonies with healthy individual bees are more likely to accumulate larger amounts of metals. This is because bees in healthy colonies are more vital. More bees go foraging and consequently more metals are accumulated. Honeybee health can be altered in different ways. Pathogens are one of the most common reasons for the decline of health of individual bees, as well as whole bee colonies (Potts et al., 2010). Another reason behind unhealthy bees and colonies can be the influence of agrochemicals, mainly pesticides. Even sub-lethal exposure to pesticides can have a negative effect on bees. It can affect their communication, as well as their lifecycle. Some pesticides can cause the shortening the life of bees up to 4 days (Wu, Anelli & Sheppard, 2011). This can influence the accumulation of metals, since these bees are a shorter time exposed to metals.

Age of the individual bees that were sampled can also have an important effect on concentrations of metals present in the honeybees. Younger bees that have not been outside of the hive are exposed to pollutants through food brought into the hive by older bees or by impact of immediate surrounding of the hive. Forager bees are bees that are 23–38 days old (Huang & Robinson, 1996). These are the bees that fly out of the hive and are able to accumulate metal pollution directly from the environment. This is the reason why mostly these bees are chosen for bioindicator studies.

### Potential effects of studied elements on human health

As can have an acute and chronic poisoning effect on humans. Acute poisoning leads to muscular pain, nausea and vomiting, abdominal pain, diarrhea, and different vascular problems that can cause circulatory collapse and kidney damage (Saha et al., 1999). Chronic exposure leads to skin lesions, and damage of internal organs, the respiratory, digestive, circulatory, neural, and renal systems. Cancer is the most significant consequence of chronic arsenic poisoning (Ng, Wang & Shraim, 2003).

Stable cesium 133 is found to be relatively safe. Some signs of mild toxicity are hypotension, gastrointestinal distress, numbness of the lips. Although it was thought that cancer cells are vulnerable to Cs there is no evidence to support this claim (Melnikov & Zanoni, 2010). Radiocesium 137 due to its emitting of beta and gama radiation is extremely hazardous even without taking it in to the body. Radioactive Cs is associated with Thyroid Cancer (Sangvanich et al., 2010).

Hg can have different effects depending on the exposure route. Inhalation of Hg vapor interstitial pneumonitis, corrosive bronchitis and may affect the nervous system. Ingestion of Hg leads to corrosive ulceration, necrosis of the gastrointestinal tract and bleeding followed by gastrointestinal damage and renal failure(Goyer & Clarkson, 2001). Methyl mercury causes neurotoxic effects in adults and toxicity to the fetuses of mothers exposed to methyl mercury during pregnancy (Bakir et al., 1973).

Mo is an essential element. The deficiency of molybdenum can cause mouth and gum disorders, hypouricemia, hyperoxypurinemia, mental disturbances, and coma. Although essential Mo can also be toxic effecting appetite, slowing growth, and causing diarrhea and anemia (Nielsen, 1996).

Many compounds containing Sb are gastrointestinal irritants that can cause nausea, vomiting, abdominal colic and diarrhea. It can also have an effect on respiratory and cardiovascular system, where it can cause myocardial damage, heart failure and cardiac arrest (Winship, 1987).

Deficiency of Se can cause Keshan disease, which is an endemic cardiomyopathy. When intake exceeds excretory capacity selenium can become toxic. Toxic effects include skin eruptions and lesions, diseased nails, and can cause different neurological symptoms (Goyer & Clarkson, 2001).

Some U salts will cause skin burns. The soluble uranium can cause acute renal damage and renal failure (Goyer & Clarkson, 2001).

Although V is thought to be an essential element no deficiency disease has been discovered. The toxicity of vanadium increases with its valence. V is mostly toxic to lungs, causing bronchitis and bronchopneumonia, but it can also cause gastrointestinal distress (Barceloux, 1999).

## Conclusions

There were statistically significant differences in metal concentrations between different months of sampling during 2014 in BG for Sb and U with higher concentrations in July. This is due to the natural lifecycle of honeybees and plants. Higher concentrations in September were observed for As and Sb in PA and Hg in PV. The reason behind this can be high participation during the peak of bee activity in spring and summer of 2014. Temporal variations were also observed in concentrations of Hg, Mo and V between the sampling years. All these elements had higher concentrations in 2014 compared to 2015. This is in accordance with the earlier findings showing that in this region metal concentrations in honeybees are in decline throughout the years.

Spatial variations were observed for Sb and U. Concentrations of Sb were statistically higher in Belgrade (BG), capitol of Serbia, compared to all other locations included in this study. Higher concentrations of Sb are attributed to intense traffic and use of antimony sulfide (Sb_2_S_2_) in manufacturing of brake linings. Uranium concentrations were significantly higher in TPP region. This region is characterized by two thermal power plants and an ash disposal site for the power plants. Considering that U is mostly retained in solid combustion waste, including ash, it can be concluded that U in this region comes from the use of coal in the thermal power plants.

This is the first study that reported concentrations of Cs and U in bodies of honeybees. For other trace elements reported in this study literature data is sparse. This study can be used as a reference for comparing concentrations of the analyzed trace elements to other regions in the world.

##  Supplemental Information

10.7717/peerj.5197/supp-1 Table S1 Average metal concentrations (mg kg^−1^) and standard deviations at five sampling locations in July and September of 2014

10.7717/peerj.5197/supp-2 Data S1 Raw data of metal concentrations for different locations, years and moths of sampling

## Additional Information and Declarations

### Data Availability

The following information was supplied regarding data availability: The raw data are provided in a Supplemental File.
