# Supplementary material for: Assessment of spatial and temporal variations in trace element concentrations using honeybees (Apis mellifera) as bioindicators
Source: PeerJ. 2018 Jul 16;6:e5197. doi: 10.7717/peerj.5197 (PMC6052853; doi:10.7717/peerj.5197)
Supplement: Table S1 [file peerj-06-5197-s001.docx]

|  | | **Element** | | | | | | | |
| --- | --- | --- | --- | --- | --- | --- | --- | --- | --- |
| **Month** | **Location** | **As** | **Cs** | **Hg** | **Mo** | **Sb** | **Se** | **U** | **V** |
| **July** | **BG** | 0.33 ± 0.18 | 0.0265 ± 0.0066 | 0.079 ± 0.031 | 0.94 ± 0.29 | 0.063 ± 0.013 | 0.26 ± 0.11 | 0.0074 ± 0.0019 | 0.249 ± 0.074 |
|  | **PA** | 0.139 ± 0.059 | 0.0235 ± 0.0079 | 0.065 ± 0.071 | 0.53 ± 0.18 | 0.0180 ± 0.0074 | 0.199 ± 0.055 | 0.00203 ± 0.00098 | 0.220 ± 0.073 |
|  | **PV** | 0.1316 ± 0.0024 | 0.0149 ± 0.0029 | 0.048 ± 0.030 | 0.61 ± 0.21 | <LOD | 0.1465 ± 0.0083 | 0.0031 ± 0.0011 | 0.164 ± 0.031 |
|  | **MS** | 0.195 ± 0.036 | 0.026 ± 0.012 | 0.026 ± 0.036 | 0.304 ± 0.024 | 0.0219 ± 0.0024 | 0.093 ± 0.022 | 0.00521 ± 0.00015 | 0.1155 ± 0.0058 |
|  | **TPP** | 0.5 ± 0.25 | 0.064 ± 0.049 | 0.052 ± 0.053 | 1.2 ± 1.3 | 0.0224 ± 0.0029 | 0.39 ± 0.15 | 0.0171 ± 0.0056 | 0.60 ± 0.33 |
| **September** | **BG** | 0.17 ± 0.049 | 0.0272 ± 0.0038 | 0.052 ± 0.035 | 0.66 ± 0.15 | 0.0318 ± 0.0011 | 0.171 ± 0.019 | 0.00179 ± 0.00079 | 0.1991 ± 0.0018 |
|  | **PA** | 0.274 ± 0.053 | 0.038 ± 0.010 | 0.141 ± 0.078 | 0.61 ± 0.25 | 0.0317 ± 0.084 | 0.293 ± 0.083 | 0.0033 ± 0.0015 | 0.289 ± 0.062 |
|  | **PV** | 0.24 ± 0.19 | 0.0118 ± 0.0025 | 0.182 ± 0.013 | 1.04 ± 0.32 | 0.023 ± 0.012 | 0.26 ± 0.12 | 0.0029 ± 0.0016 | 0.222 ± 0.083 |
|  | **MS** | - | - | - | - | - | - | - | - |
|  | **TPP** | 0.25 ± 0.13 | 0.038 ± 0.015 | 0.035 ± 0.016 | 0.515 ± 0.052 | 0.0182 ± 0.0033 | 0.383 ± 0.099 | 0.0125 ± 0.0074 | 0.45 ± 0.36 |

Supplementary Table 1. Average metal concentrations (mg kg^-1^) and standard deviations at five sampling locations in July and September of 2014.
